# Drug Utilization Study of Gastroprotective Agents in Medicine and Surgery Wards of a Tertiary Care Teaching Hospital

**DOI:** 10.7759/cureus.33739

**Published:** 2023-01-13

**Authors:** Harsh Koyani, Neel Vora, Mitul Kalathia, Nihar Patel, Samidh Shah

**Affiliations:** 1 Internal Medicine, Byramjee Jeejeebhoy (BJ) Medical College, Ahmedabad, IND; 2 Pediatrics, Shantabaa Medical College, Amreli, IND; 3 Pharmacology, Byramjee Jeejeebhoy (BJ) Medical College, Ahmedabad, IND

**Keywords:** adverse drug reactions, gastroprotective agents, co-prescription, drug interactions, prescribing patterns, proton pump inhibitors, drug utilization research

## Abstract

Introduction

Drug utilization research (DUR) is “the marketing, distribution, prescription, and use of drugs in a society, with special emphasis on the resulting medical, social, and economic consequences” as per the definition of the World Health Organization (WHO). The ultimate goal of DUR is to evaluate whether the drug treatment is rational or not. Various gastroprotective agents are available today, such as proton pump inhibitors, antacids, and histamine 2A receptor antagonists (H2RAs). Proton pump inhibitors block the gastric H+/K+-adenosine triphosphatase (ATPase) via covalent binding to cysteine residues of the proton pump to inhibit gastric acid secretion. Antacids are compounds containing different combinations, such as calcium carbonate, sodium bicarbonate, aluminum, and magnesium hydroxide. Histamine 2A receptor antagonists (H2RAs) decrease gastric acid secretion by reversibly binding to histamine H2 receptors located on gastric parietal cells, thereby inhibiting the binding and action of the endogenous ligand histamine. A recent literature review has shown that inappropriate use of gastroprotective agents has increased the risk for adverse drug reactions (ADRs) and drug interactions.

Methods

A total of 200 inpatient prescriptions were analyzed. The extent of prescribing, dosing information given, and cost incurred on the use of gastroprotective agents in both surgery and medicine inpatient departments was assessed. Prescriptions were also analyzed using WHO core indicators and for drug-drug interactions.

Results

Proton pump inhibitors were prescribed to 112 male patients and 88 female patients. The most common diagnosis was diseases of the digestive system with 54 (27.5%) cases, followed by diseases of the respiratory tract with 48 (24%) cases. Out of 200 patients, 51 comorbidities were reported from 40 patients. Among all prescriptions, injection of pantoprazole was the most common route of administration (181 (90.5%)), followed by tablets of pantoprazole (19 (9.5%)). The most common dose prescribed of pantoprazole was 40 mg in 191 (95.5%) patients in both departments. The frequency of therapy was also most commonly prescribed twice daily (BD) in 146 (73%) patients. Potential drug interaction was most commonly found with aspirin in 32 (16%) patients. The total cost incurred on proton pump inhibitor therapy of the medicine and surgery departments was 20,637.4 Indian Rupees (INR). Of this, the cost for patients admitted in the medicine ward was 11,656.12 INR and in the surgery department was 8,981.28 INR.

Conclusion

Gastroprotective agents are a group of drugs that are used to protect the stomach and the gastrointestinal tract (GIT) from acid-related injury. Our study found that proton pump inhibitors were the most commonly prescribed gastroprotective agents among inpatient prescriptions, with pantoprazole being the most frequently used. The most common diagnosis among patients was diseases of the digestive system, and most of the prescriptions were for injection administration at a dose of 40 mg twice daily. These findings suggest that there may be opportunities for improvement in the rational use of gastroprotective agents to reduce the risk of adverse drug reactions and interactions and lower healthcare costs. Overall, the study highlights the need for healthcare providers to be aware of the appropriate use of gastroprotective agents to minimize irrational prescriptions and reduce polypharmacy.

## Introduction

Drug utilization research (DUR) is defined as “the marketing, distribution, prescription, and use of medications in a society, with particular emphasis on the resulting medical, social, and economic repercussions” by the World Health Organization (WHO) [1]. The main objective of DUR is to determine the rationality of drug treatment, which may offer insights into numerous elements of prescribing patterns of drugs with frequency, dose, length of therapy, indication, and outcomes of drug usage. In both outpatient and inpatient settings, gastroprotective agents are among the most frequently prescribed classes of drugs. Antacids, histamine 2A receptor antagonists (H2RAs), proton pump inhibitors, and cytoprotective drugs such as misoprostol are examples of gastroprotective drugs [2]. Proton pump inhibitors are most commonly co-prescribed with nonsteroidal anti-inflammatory drugs (NSAIDs) and/or antimicrobial agents (AMAs) because of their gastroprotective effect [3].

Studies have shown that the incidence of irrational use of proton pump inhibitors ranges between 40% and 70% [4]. Proton pump inhibitors inhibit stomach acid secretion by blocking the gastric H+/K+-adenosine triphosphatase (ATPase) through covalent binding to proton pump cysteine residues (omeprazole, lansoprazole, pantoprazole, rabeprazole, and esomeprazole). They are used in the management of gastroesophageal reflux disease (GERD), upper gastrointestinal bleeding (including varices), Barrett’s esophagus, Zollinger-Ellison syndrome, ulcer healing, *Helicobacter pylori* eradication, and prophylaxis of peptic ulcer disease, and these are among the common indications for proton pump inhibitor prescription that are recommended by the National Institute for Health and Care Excellence (NICE) guidance [5].

A recent review of the literature has shown that inappropriate use of gastroprotective agents has increased the risk for adverse drug reactions (ADRs) and drug interactions. These agents are being overutilized because of easy availability, high efficacy, competitive marketing, and expanded indications. Constipation, headache, abdominal pain, flatulence, and diarrhea are all examples of the mild and self-limiting side effects of gastroprotective agents. The long-term repercussions include an increased risk of hip fractures, gastric carcinoids, hypomagnesemia, and *Clostridium difficile* infection [6]. Few serious ADRs such as cardiac arrest have also been reported due to ranitidine probably because of medication errors [7].

The fundamental step to limiting the irrational use of medicines is to quantify the extent to which this is occurring. In the 1990s, the WHO in collaboration with the International Network for Rational Use of Drugs (INRUD) developed a set of indicators to measure the performance of healthcare facilities related to the utilization of drugs. The prescribing indicators include the average number of drugs prescribed per encounter, the percentage of drugs prescribed by generic name, the percentage of encounters where an antibiotic was prescribed, the percentage of encounters where an injection was the route of administration, and the percentage of drugs prescribed from the Essential Drugs List (EDL) [8].

The present study was designed to assess and evaluate the utilization patterns of gastroprotective agents in patients admitted to the medicine and surgery departments as these departments are burdened by one of the largest patient flow of any tertiary care teaching hospital. Our study aims to provide new insights into the drug utilization patterns of gastroprotective agents as this is one of the few studies to evaluate it in the medicine and surgery departments. The majority of the prior studies we came across were in the orthopedics department based on our review of the literature.

## Materials and methods

This was a prospective observational study carried out over a period of two months (May to June). Study approval was taken from the Institutional Ethics Committee of Civil Hospital Ahmedabad (CHA) (reference number EC/Approval/59/2021/01/06/2021). Written permission was taken from the Head of the Department of Medicine and Surgery of CHA. The principal investigator visited both departments daily. Patients were enrolled as per inclusion criteria, and all necessary information was collected and recorded in a pretested case record form (CRF), which included demographic details, prescription patterns of gastroprotective agents, other details of comorbidities, diagnosis of patients, and other concomitant drugs prescribed. Data regarding the dose of individual drugs, their dosage form, frequency and route of administration, and total days of therapy were also recorded. A cost analysis of gastroprotective agents was done, and the average cost per patient for gastroprotective agents was calculated. The patients were followed up until discharge. Prescriptions of both departments were evaluated according to WHO core indicators for prescriptions. Data were analyzed using descriptive statistics. Details such as gender, diagnosis of patients, and prescription pattern of gastroprotective agents were analyzed using frequency and percentage. Mean and standard deviation were used to consider the age and average cost of gastroprotective agents per patient, which was spent in Indian Rupees (INR).

The inclusion criteria for subject selection were patients of any gender above 18 years of age who are hospitalized, with a minimum of one gastroprotective agent prescribed during hospitalization, and patients who were willing to give written informed consent. The exclusion criteria were patients not receiving gastroprotective agents during hospitalization and patients not willing to give written informed consent.

Patients were selected using the simple random sampling method. The sample size of the study was 200 (100 each from medicine and surgery departments). The collected data were analyzed as per the following headings: indication for the prescription of gastroprotective agents, type of formulation prescribed, duration of therapy, total number of gastroprotective agents prescribed, drug-drug interactions (drug-drug interaction was checked using the Medscape drug interaction checker and drug interaction checker from drugs.com), and cost analysis of gastroprotective agents.

Drug interactions can fall into three categories: minor (minimally clinically significant; minimize risk, assess risk and consider an alternative drug, take steps to circumvent the interaction risk, and/or institute a monitoring plan (seen with aspirin)), moderate (moderately clinically significant; usually avoid combinations and use it only under special circumstances (seen with amphotericin b, atorvastatin, dabigatran, furosemide, levothyroxine, iron, warfarin, and clopidogrel)), and major (highly clinically significant; avoid combinations; the risk of the interaction outweighs the benefit) [9].

## Results

Demographic details of the study subjects 

In total, 200 patients were enrolled, 100 each from the medicine and surgery departments. The mean age of the patients was 43.05 ± 16.18 years. The mean age of patients in the medicine department was 45.29 ± 15.91 years and in the surgery department was 41.54 ± 15.86 years. In both these departments, the majority of the patients belonged to the age group of 30-44 years (medicine: 39%, surgery: 38%). The majority of the patients were male in both medicine (n = 54) and surgery (n = 58) departments, whereas the number of female patients was 46 and 42 in the medicine and surgery departments, respectively.

Distribution of comorbidities in patients

Out of 200 patients, 51 comorbidities were reported from 40 patients, with the most common comorbidity being diabetes (21 (41.17%)), followed by hypertension (14 (27.45%)) and ischemic heart disease (5 (9.8%)).

Diagnosis of the patients

The diagnosis of the patients was classified according to the International Classification of Diseases 11th Revision (ICD-11). The most common diagnosis was diseases of the digestive system (54 (27.5%)), followed by diseases of the respiratory tract (48 (24%)) (Table 1).

**Table 1:** Diagnosis of patients

Diagnosis according to the International Classification of Disease	Number of patients (n = 200) (%)	Medicine department	Surgery department
Diseases of the digestive system	54 (27%)	14	40
Diseases of the respiratory system	48 (24%)	35	13
Symptoms, signs, or clinical findings, not elsewhere classified	11 (5.5%)	6	5
Diseases of the nervous system	12 (6%)	12	-
Diseases of the genitourinary system	10 (5%)	7	3
Diseases of the blood or blood-forming organs	10 (5%)	6	4
Injury, poisoning, or certain other consequences of external causes	17 (8.5%)	2	15
Certain infectious or parasitic diseases	3 (1.5%)	2	1
Diseases of the circulatory system	21 (10.5%)	10	11
Endocrinal disorders	11(5.5%)	6	5
Neoplasms	3 (1.5%)	-	3

Evaluation of prescription by WHO core indicators

All 200 prescriptions were evaluated according to WHO core indicators for prescriptions. The average number of drugs prescribed per prescription was 7.55. Out of 200 prescriptions, 92.5% were encountered with antibiotics, and 95.5% of the prescriptions had injectables. The further individual department-wise breakup of core indicators is shown in Table 2.

**Table 2:** Evaluation of prescriptions by WHO core indicators WHO: World Health Organization

WHO indicators	Overall (n = 200)	Medicine (n = 100)	Surgery (n = 100)
Average number of drugs per encounter	7.55	7.88	7.08
Percentage of encounters with antibiotics	92.5%	94%	91%
Percentage of encounters with injection	95.5%	98%	93%
Percentage of drugs prescribed by generic name	70.58%	74.49%	66.24%
Percentage of drugs from the essential drug list	83.81%	81.05%	86.58%

Duration of the use of gastroprotective agents in the number of days

Out of the 200 subjects enrolled, the majority (123 (61.5%)) received pantoprazole for 1-7 days, followed by 8-14 days in 52 (26%) patients and 15-21 days in 14 (7%) patients. The mean duration for which pantoprazole was prescribed in the medicine department was 9.49 ± 6.55 days and in the surgery department was 7.12 ± 6.49 days.

Different routes of administration of gastroprotective agents

Out of the total 200 cases, the majority (181 (90.5%); medicine: 95, surgery: 86) received pantoprazole by intravenous (IV) route, followed by oral form (19 (9.5%); medicine: 5, surgery: 14).

Frequency of gastroprotective agent prescription

The most commonly prescribed dosage frequency was twice daily dose (BD) (146 (73%)), followed by once daily dose (43 (21.5%)) and thrice daily dose (10 (5%)). Only one (0.5%) patient received pantoprazole four times a day.

Cost analysis of gastroprotective agents

Cost analysis was performed by knowing the per unit price of formulations supplied by the Gujarat government to the hospital pharmacy. The per unit cost of pantoprazole injection was 7.40 INR, and for tablet pantoprazole, it was 0.52 INR. The average per-patient cost for intravenous gastroprotective agents was 121.98 INR for the medicine department and 103.68 INR for the surgery department, whereas for oral route, it was 13.62 INR for the medicine department and 4.59 INR for the surgery department.

Potential drug interactions of gastroprotective agents

Out of 200 cases, we were able to find 87 potential drug interactions with gastroprotective agents among 76 patients. The most common interaction found was with aspirin (32 (16%)), followed by dabigatran and furosemide (18 (9%) each). However, 100% of the evaluated possible interactions were of the moderate category. We could not find any minor or major drug interactions in our study.

Co-prescription with antimicrobials

**Figure 1:**
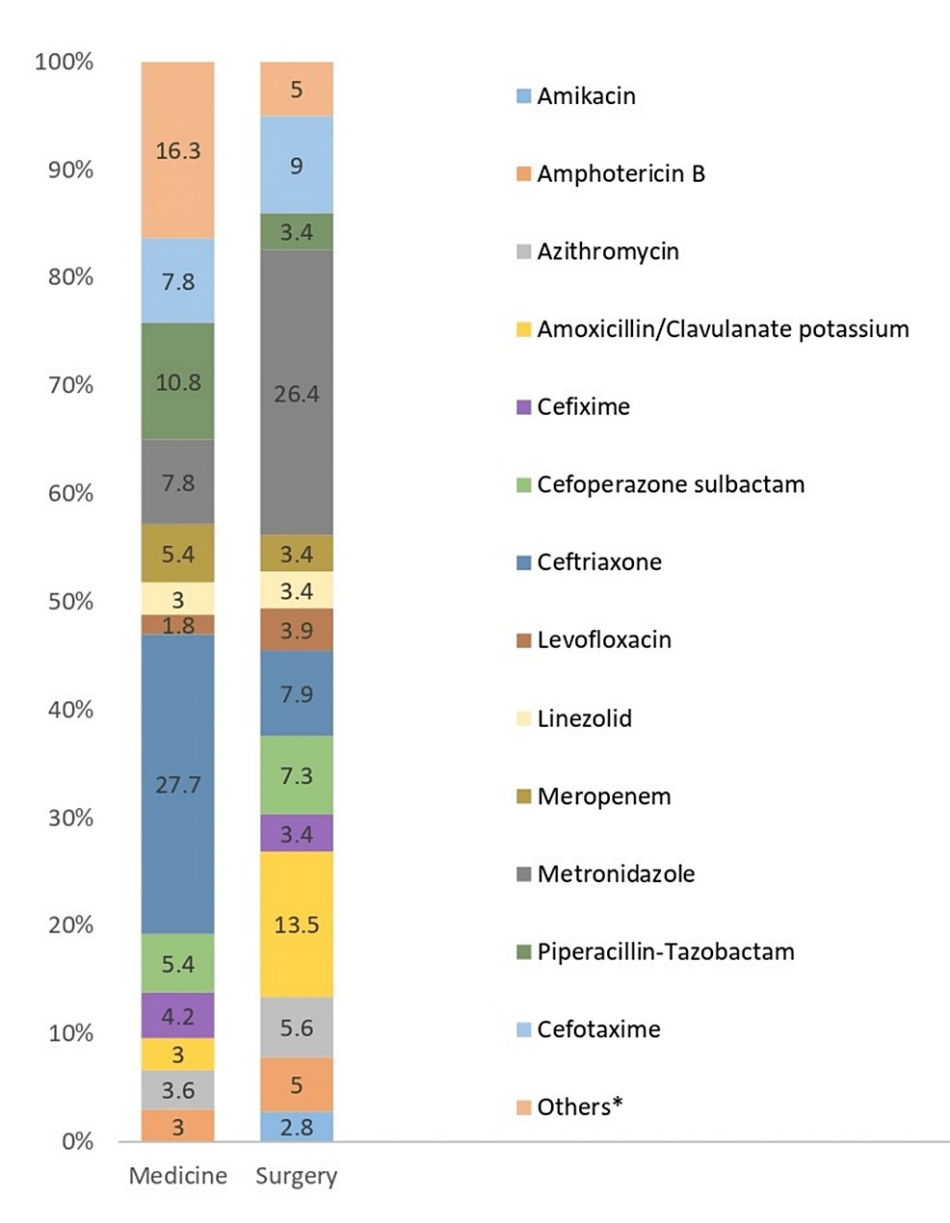
Co-prescription with antimicrobials *Other antimicrobials include acyclovir; ciprofloxacin; ofloxacin; fluconazole; isoniazid, rifampin, pyrazinamide, and ethambutol (HRZE); nitazoxanide; rifaximin; trimethoprim/sulfamethoxazole; tigecycline; voriconazole; doxycycline; moxifloxacin; penicillin; itraconazole; posaconazole; remdesivir; and gentamycin.

In total, 344 antimicrobials were prescribed to 200 patients. The most commonly prescribed antimicrobial in the surgery ward was metronidazole (medicine: 7.8%, surgery: 26.4%), followed by amoxicillin/clavulanate (medicine: 3%, surgery: 13.5%) and ceftriaxone (medicine: 27.7%, surgery: 7.9%), and piperacillin/tazobactam (medicine: 10.8%, surgery: 3.4%) was the commonly prescribed antimicrobials in medicine wards (Figure 1).

Co-prescription with analgesics

**Figure 2:**
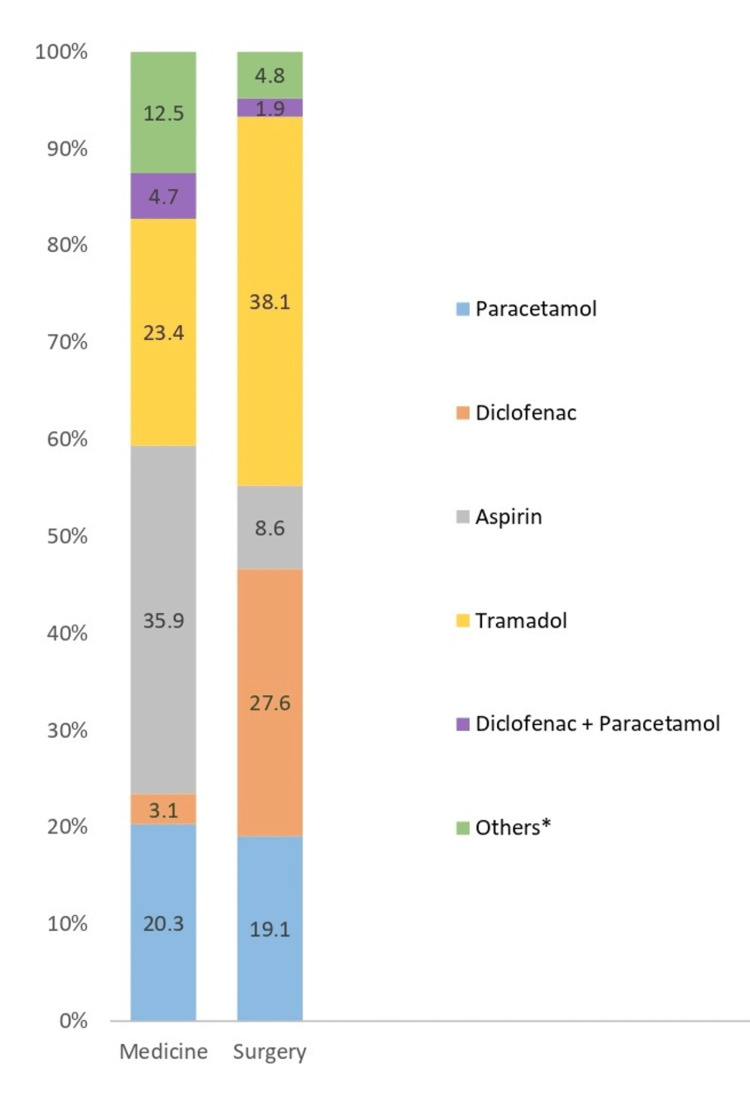
Co-prescription with analgesics *Others include drugs prescribed rarely, which include the following drugs: ibuprofen/paracetamol, tramadol + acetaminophen, naproxen, indomethacin, and mefenamic acid.

A total of 169 analgesics were prescribed. Aspirin (medicine: 35.9%, surgery: 8.6%) and tramadol (medicine: 23.4%, surgery: 38.1%) were more commonly prescribed in the medicine wards, while tramadol (medicine: 23.4%, surgery: 38.1%) and diclofenac (medicine: 3.1%, surgery: 27.6%) were more commonly prescribed in the surgery ward (Figure 2).

## Discussion

DUR studies are crucial for both the care of individual patients and for policy-making at the national level. The availability of information on drug use is however insufficient in the majority of middle- or low-income countries [10].

In this study, we analyzed 200 prescriptions of patients admitted to the indoor departments of surgery and medicine wards (100 each) of a tertiary care teaching hospital. All 200 prescriptions had proton pump inhibitors prescribed, among which pantoprazole was prescribed in all patients. Most patients received pantoprazole via intravenous route (medicine: 95%, surgery: 86%), followed by oral route (medicine: 5%, surgery: 14%). The mean duration for which pantoprazole was prescribed in the medicine department was 9.49 ± 6.55 days and in the surgery department was 7.12 ± 6.49 days. Most prescriptions had a dosage frequency of twice daily (BD) dose (medicine: 71%, surgery: 75%). The most common dose used was 40 mg in 90.5% of patients.

In our study, in both departments, the most common age group was between 31 and 45 years (38%). A similar study conducted in 2017 showed that most of the patients belonged to the age group of 41-60 years old (146, 42.8%), followed by patients above 60 years old (111, 32.6%), and the remaining patients were of age 18-40 years (84, 24.6%) [11].

Of the 200 patients in our study, 40 (20%) had comorbidities, with the most common comorbidity being diabetes mellitus, followed by hypertension. As comorbidity increases, the number of drugs prescribed also increases, which in turn leads to increased use of proton pump inhibitors. This is further reinforced by the fact that of all patients, the majority had disorders related to the gastrointestinal tract (GIT), which leads to an increasing number of prescriptions for proton pump inhibitors. Overall, multiple factors contribute to the increasing number of proton pump inhibitor prescriptions including comorbid conditions, primary diagnosis pertaining to the GIT, and concurrent prescription of antimicrobials and NSAIDs.

Analysis of prescriptions using WHO core indicators shows that polypharmacy is observed in most of the patients. Polypharmacy, defined as the regular use of at least five medications, is common in older adults and younger at-risk populations and increases the risk of adverse medical outcomes [12]. As the number of drugs prescribed increases, it directly correlates to the increasing cost of drug therapy, hence posing a greater financial burden on the patient. Moreover, with a higher number of drugs prescribed, there could be a higher number of drug-drug interactions, which can complicate drug therapy and might also cause the failure of drug therapy in some cases. The physician should be aware of these disadvantages of polypharmacy. We have also observed that almost all prescriptions have antibiotics, which is unnecessary and leads to irrational use, promoting antimicrobial drug resistance. The same was observed for injectable drug preparations, which increase the cost to the patient and government and also puts the patients at risk of developing ADRs. Similar findings were observed by Kaur et al. [13] in 2014 in North India.

All 200 prescriptions had proton pump inhibitors prescribed, and among them, pantoprazole was prescribed in 100% of the prescriptions. Almost similar findings were observed by Lincy et al. [14] and Tadvi et al. [4], where pantoprazole was the most common proton pump inhibitor prescribed in 83% of cases. This shows that pantoprazole is preferred to other gastroprotective agents because of its efficacy, safety, and lesser cost.

Most patients received pantoprazole via intravenous route in our study. Similar results were observed by Verma et al. [15] in 2019, where 80% received pantoprazole via intravenous route. One of the reasons for the intravenous route being the preferred choice is the faster onset of action as compared to the oral route. Furthermore, both studies were done in indoor patients, which may also explain the choice of route of administration. However, evidence suggests that even oral therapy is highly effective, similar in effectiveness to intravenous (IV) proton pump inhibitors at equivalent doses, and actually, only a small proportion of patients with upper GIT bleeding require the parenteral use of proton pump inhibitors [16]. Therefore, there is a need for developing institutional protocols and conducting training programs for emphasizing appropriate prescribing practices with regard to parenteral proton pump inhibitors.

The mean duration for pantoprazole prescribed in the medicine department was 9.49 ± 6.55 days and in the surgery department was 7.12 ± 6.49 days. The duration of proton pump inhibitor therapy generally varies from eight to 12 weeks in GERD patients, seven to 14 days in *Helicobacter pylori* eradication, four to eight weeks in dyspepsia, and eight weeks in gastroduodenal lesions [17]. Either less or more duration can lead to failure of therapy and cost to the patient, which is why the duration of proton pump inhibitors should be appropriate.

In our study, most prescriptions included a twice daily dose of pantoprazole (medicine: 71%, surgery: 75%). However, the defined daily dose for pantoprazole is 40 mg once a day as proposed by the WHO [18]. A possible reason for this difference may be that proton pump inhibitors can be given twice daily to achieve steady-state concentration as reported by Tadvi et al. [4], which is not acceptable as, although the plasma half-life is short, its action lasts longer [19].

Drug-drug interactions are common when there is polypharmacy. This can lead to sometimes fatal ADRs, thereby marking the importance to check for drug-drug interactions when multiple drugs are prescribed. In our study, the major interactions found with proton pump inhibitors were with aspirin (32 (16%)), followed by dabigatran (18 (9%)), furosemide (18 (9%)), and atorvastatin (17 (8.5%)). Proton pump inhibitors and H2 blockers alter the gastric pH and may affect the pharmacokinetics of drugs. Most of our observations are moderate in nature. Similar observations were noted in a study conducted by Goyal et al. [20] in 2020 and Patel et al. [21] in 2016. Although these were theoretical observations, sometimes, they can lead to serious drug interactions, causing patient morbidity and mortality.

In our study, we have seen that patients were also receiving antimicrobials and NSAIDs, which are known to cause GIT side effects, and to prevent them, pantoprazole was prescribed, which however is not justifiable.

Our findings also show that the cost of intravenous pantoprazole ranged from 14 INR to 124 INR, while the cost of tablet form ranged from 4 INR to 13 INR. The cost is an important factor in deciding the drug therapy since it can affect patient compliance and outcomes. Proton pump inhibitors are more expensive than other acid-suppressant agents. Hence, inappropriate prescription of proton pump inhibitors is of concern more so in resource-limited settings. The majority of patients may not need gastroprotective agents, and these costs can be minimized by applying the principles of rational drug therapy.

The limitations of our study include a small sample size of 200 patients, the study being conducted in a single center, analyzing only inpatient prescriptions for the evaluation of drug utilization of gastroprotective agents, and that our study was limited to only the medicine and surgery departments.

## Conclusions

Despite the above limitations, our study leads to important findings. We have observed that all prescriptions contained antimicrobials and injectable drug preparations. We have also observed that polypharmacy was present in our study, and there was an inappropriate use of pantoprazole in terms of dose, frequency, and duration. As noted by our findings that there is excessive but avoidable use of antimicrobials, we recommend that there should be a drug therapeutic committee in each hospital to keep the use of antimicrobials in check.

We also observed that the majority were prescribed by their generic name and from the EDL. This shows that the prescriber is aware of the importance of generic names and the EDL. However, we suggest that the prescriber should be made aware of the disadvantages of polypharmacy, irrational use of antibiotics, and injectable drug preparations. The study of knowledge awareness and practice for this can be done, and according to that, further training can be given to them.

The prescriber should also be made aware of the cost of therapy and possible drug-drug interactions. The same can be included in teaching undergraduate students so that future prescribers can have the knowledge of the same.

## Appendices

Table 3 shows the case report form used in the present study.

**Table 3:** Case report form

Patient name:																		Serial number:						Indoor number:					
Gender (M/F):						Age:									Unit:						Department:								
Occupation:															MRD number:						Income/month:								
Date of admission:															Date of discharge:														
Chief complaints																													
Presenting complaints:																													
Provisional diagnosis:																													
Final diagnosis:																													
Personal history of:															Smoking/tobacco (Y/N):														
																														Alcohol consumption (Y/N):														
															Appetite:																													
																														Bladder/bowel habits:														
															Allergic reactions to drugs and food:																													
																														Past history of:															Surgery:														
															Tuberculosis:																																												
																																													Diabetes mellitus:														
															Hypertension:																																												
																																													Treatment:																													
Serial number:			Generic name			Date						Prescribed dose			Route			Frequency			Supply			Cost			Drug interaction																	
						From			To																																			
Adverse drug event:																																												
System organ class (SOC):																													
Suspected drug:																													
Total cost of therapy of gastroprotective agents:																													
WHO core prescribing indicators for audit:																													
Serial number:										Indicators										Number									
1										Number of drugs prescribed																			
2										Number of drugs prescribed by generic name																			
3										Number of antibiotics prescribed																			
4										Number of injections prescribed																			
5										Number of drugs prescribed from formulary																			
